# *w*Mel *Wolbachia* genome remains stable after 7 years in Australian *Aedes aegypti* field populations

**DOI:** 10.1099/mgen.0.000641

**Published:** 2021-09-01

**Authors:** Kimberley R. Dainty, Jane Hawkey, Louise M. Judd, Etiene C. Pacidônio, Johanna M. Duyvestyn, Daniela S. Gonçalves, Silk Yu Lin, Tanya B. O'Donnell, Scott L. O'Neill, Cameron P. Simmons, Kathryn E. Holt, Heather A. Flores

**Affiliations:** ^1^​ Institute of Vector-Borne Disease, Monash University, Melbourne, Victoria, Australia; ^2^​ Department of Microbiology, Monash University, Melbourne, Victoria, Australia; ^3^​ Department of Infectious Diseases, Central Clinical School, Monash University, Melbourne, Victoria, Australia; ^4^​ World Mosquito Program, Monash University, Melbourne, Victoria, Australia; ^5^​ Oxford University Clinical Research Unit, Hospital for Tropical Diseases, Ho Chi Minh City, Vietnam; ^6^​ London School of Hygiene and Tropical Medicine, London WC1E 7HT, UK

**Keywords:** *Aedes aegypti*, genome evolution, *Wolbachia*

## Abstract

Infection of *w*Mel *Wolbachia* in *Aedes aegypti* imparts two signature features that enable its application for biocontrol of dengue. First, the susceptibility of mosquitoes to viruses such as dengue and Zika is reduced. Second, a reproductive manipulation is caused that enables *w*Mel introgression into wild-type mosquito populations. The long-term success of this method relies, in part, on evolution of the *w*Mel genome not compromising the critical features that make it an attractive biocontrol tool. This study compared the *w*Mel *Wolbachia* genome at the time of initial releases and 1–7 years post-release in Cairns, Australia. Our results show the *w*Mel genome remains highly conserved up to 7 years post-release in gene sequence, content, synteny and structure. This work suggests the *w*Mel genome is stable in its new mosquito host and, therefore, provides reassurance on the potential for *w*Mel to deliver long-term public-health impacts.

## Data Summary

Raw sequence data and assembled genomes are available from the National Center for Biotechnology Information (NCBI) under BioProject accession number PRJNA695307.

**Impact Statement**

*Aedes aegypti* mosquitoes transmit a number of arboviruses that cause human disease, including dengue. The introduction of the *w*Mel strain of *Wolbachia* into *Ae. aegypti* populations has proven to be an effective biocontrol method for dengue in Cairns, Australia, where it was first established in 2011. The infection of *w*Mel into *Ae. aegypti* significantly reduces the ability of the mosquito to transmit virus between humans. It also causes a reproductive manipulation that enables introduction of *w*Mel into wild-type mosquito populations. These two features must remain stable in field conditions for the continued success of this intervention. Here, we examine the genomic evolution of *w*Mel in *Ae. aegypti* since its establishment in Cairns, Australia. By using two sequencing methods, we are able to examine the gene sequence, content and structure. We find that the *w*Mel genome has remained highly conserved in *Ae. aegypti*, since its establishment in Cairns, Australia. This work gives reassurance on the long-term applicability of *w*Mel as a biocontrol method for arboviruses such as dengue.

## Introduction

Dengue is the fastest growing mosquito-borne disease, having increased in incidence by 30-fold over the past 50 years [1]. Around 400 million people a year are estimated to be infected by dengue viruses, with over half of the world’s population at risk of the disease [2]. As historical methods to control arboviral disease transmission such as chemical insecticide-based programmes and breeding-site reduction struggle to compete with increasing urbanization, population density and global transportation systems, novel vector control methodologies are being developed to address this growing problem [3, 4].

A group of novel vector control methodologies focus on the use of the bacteria, *Wolbachia pipientis. Wolbachia* is a maternally inherited endosymbiont present in 40–70 % of insects worldwide [5–7]. Many *Wolbachia* strains induce a reproductive manipulation called cytoplasmic incompatibility (CI). This provides a fitness advantage to *Wolbachia *-infected females, helping to drive *Wolbachia* into wild-type populations [8, 9]. More recently, multiple *Wolbachia* strains have been shown to provide host protection from pathogenic viruses [10–12]. The World Mosquito Program (WMP) transinfected a strain of *Wolbachia* native to *Drosophila melanogaster*, *w*Mel, into *Aedes aegypti*. This infection causes the two desired features, CI [13, 14] and inhibition of arbovirus transmission [13, 15–22], which underpin the biocontrol method.

*w*Mel-infected *Ae. aegypti* were first released in Australia in the Cairns suburbs of Gordonvale and Yorkeys Knob in 2011. After 10 weeks of releases, *w*Mel was successfully introgressed into the wild-type population and has remained at high frequencies since [23–26]. *w*Mel-infected *Ae. aegypti* populations have since been established throughout the regional cities of Cairns, and other regional communities with histories of dengue outbreaks. Throughout this time, the region has seen a cessation in local dengue transmission [26, 27]. However, continued success of this *Wolbachia* intervention relies upon these desired *Wolbachia *-induced phenotypes (CI and pathogen blocking) remaining stable in field *Ae. aegypti* populations.

Prior to establishing a stable *Wolbachia* infection in *Ae. aegypti, w*Mel was first transfected from *D. melanogaster* into the mosquito cell line RML12. It was thought this would provide *w*Mel with an opportunity to adapt to a mosquito cell environment and increase the likelihood of successful transinfection into *Ae. aegypti* [13]. The impact of the *D. melanogaster* > RML12 > *Ae. aegypti* host transfers on the *w*Mel genome is not well understood. Since the initial sequencing of *w*Mel directly from its native *D. melanogaster* host in 2004 [28], only one study has sequenced the *w*Mel genome in *Ae. aegypti*. The study showed little variation in the sequenced genomes compared to the reference genome. However, due to limited sampling and sequencing methods, the impact of host transfers and field establishment is still not fully understood [29]. A strain of *Wolbachia* closely related to *w*Mel, *w*MelPop, underwent a similar passage into *Ae. aegypti*, via two cell lines. Sequencing and analysis of this strain after cell line adaptation revealed a number of changes including an IS*5* insertion, a multi-gene deletion and a small number of SNPs. No further changes were observed in *w*MelPop after transinfection into *Ae. aegypti*; however, sequencing occurred only a short time after transinfection [30].

The *w*Mel genome contains high levels of repetitive DNA, mobile genetic elements and three prophage sequences – two *Wolbachia* prophage (phage WO) and one pyocin-like element [28]. Large expansion or movements of prophage WO have been identified in other *Wolbachia* genomes [31, 32]. The mechanism of *w*Mel-induced viral inhibition and what *w*Mel gene(s) may underlie this induced phenotype are unknown. However, the induction of CI has been attributed to two genes within the WO prophage [33, 34]. Therefore, this prophage is a region of particular interest, as CI is essential for *Wolbachia* introgression and sustainability in field populations.

Many endosymbionts including *Wolbachia* are maternally transmitted in insects. This method of transfer results in a significantly lower population size in embryos compared with adults [35]. The evolutionary consequences of population bottlenecks depend on the severity of the given bottleneck, with narrow bottlenecks having been suggested to influence the evolutionary dynamics of insect symbionts as a result of changes in the efficacy of purifying selection [36, 37]. As the severity of population reduction experienced by *Wolbachia* has not been studied, implications as to this effect are unknown. However, studies of the geographical haplotype structuring of *w*Mel in its native host *D. melanogaster* have calculated a low mean genome-wide nucleotide diversity across populations [38, 39]. As the *w*Mel-infected colony of *Ae. aegypti* was produced from transinfection of a single female [13], this strong bottleneck likely severely impacted the variation of *w*Mel within the *Ae. aegypti*.

This study assessed the genomic changes to the *w*Mel genome after its transinfection into the novel *Ae. aegypti* host. Sequencing was performed on *w*Mel from *Ae. aegypti* collected from release areas in Cairns, Australia, in both 2011 and 2018. The *w*Mel genome sequence from 2011 was compared to the *w*Mel reference genome from *D. melanogaster* to identify changes accrued during host transfers, while genomes from 2018 were compared to those from 2011 to identify changes that have occurred post-field release. Through a combination of short- and long-read sequencing, we show that the *w*Mel genome has remained highly stable despite multiple host transfers, and 7 years post-release in *w*Mel-infected mosquitoes in the field. This study strengthens a growing portfolio of evidence supporting the effectiveness and stability of *Wolbachia* introgression as a public-health intervention.

## Methods

### Sample collection and processing

Samples from 2011 were acquired via ovitraps deployed March–April 2011. *Ae. aegypti* eggs were collected from Gordonvale, Cairns. Eggs were hatched at 26 °C, 60 % relative humidity with a 12 h light:dark cycle, and reared to second instar larvae before being stored in 80 % ethanol at 4 °C. A total of 10 larval samples were sequenced (Table S1, available with the online version of this article).

Samples from 2018 were acquired via ovitraps deployed March–May 2018. *Ae. aegypti* eggs were collected from four release suburbs in Cairns (Gordonvale, Yorkeys Knob, Mount Sheridan, Smithfield). Eggs from each ovitrap were hatched and reared at 26 °C, 60 % relative humidity with a 12 h light:dark cycle. Mosquitoes were aged at least 7 days post-emergence when their ovaries were obtained to enrich for *Wolbachia *, as *w*Mel density is substantially higher in this tissue [10]. Ovaries from 2 to 30 *Ae. aegypti* females per trap were dissected and frozen in liquid nitrogen before being stored at −80 °C. As ovitraps are likely to contain full-sibling individuals [40, 41], each of these collections are considered to represent the offspring from a single *Wolbachia-*infected mother. A total of 30 samples across the four suburbs were sequenced (Table S1).

Samples were homogenized in buffer ATL using a hand-pestle, before DNA was extracted using the MagAttract HMW DNA kit (Qiagen) with a 50 µl elution in nuclease-free water, following the manufacturer’s instructions. Libraries were prepared for each sample with the Nextera DNAFlex library prep kit (Illumina) using unique index tags. Nextera DNAFlex libraries were prepared according to the manufacturer’s directions with one significant deviation, all reactions were scaled to 25 % of recommended volumes. This change did not significantly affect the performance of the library preparation. Samples were sequenced via Illumina platforms, generating 150-base-paired-end reads. Details regarding samples and associated sequencing data are available in Table S2.

For long-read sequencing, library preparation was performed using the ligation-based kit (LSK109) with native barcoding (NBD103) to multiplex samples (Oxford Nanopore Technologies), before being loaded onto a R9.4 flow cell (Oxford Nanopore Technologies) and sequenced using the MinION device (Oxford Nanopore Technologies), as described previously [42]. DNA was not sheared or size selected prior to library preparation. Reads were basecalled with Guppy v3.6.0 using the R9.4.1 450bps HAC (high accuracy) model. Reads were filtered using Filtlong (https://github.com/rrwick/Filtlong) using default parameters to keep reads at least 1000 bp long before further analysis. Details regarding samples and associated sequencing data are available in Table S2.

### Analysis

BioBloom Tools v2.3.2 [43] was used to identify Illumina reads belonging to *Ae. aegypti* (accession no. NC_035107.1) using default parameters, and these reads were excluded from downstream analysis. Single nucleotide variants were identified via mapping the remaining Illumina reads to the *D. melanogaster w*Mel reference genome [28] (NC_002978.6) using the RedDog v1b.11 pipeline (https://github.com/katholt/reddog) according to the developers’ guidelines and using standard parameters. Reads were not trimmed prior to analysis. Briefly, Bowtie2 version 2.2.3 [44] was used to map reads to the reference sequence, before SAMtools version 1.9 [45] called SNPs with QUAL values ≥30. For 2011 samples, Illumina read depth varied between 16.58× and 97.78× (mean 41.84×). Coverage was 100 % for 9/10 genomes, and 99.99 % for the remaining sample. For 2018 samples, depth ranged between 73.17× and 448.18× (mean 179.69×), with coverage of 100 % achieved for all genomes. Visualization of the polymorphisms across the genome was created using CiVi [46] . Low-frequency variants were detected in samples from 2018 with LoFreq v2.1.3.1 [47] using standard parameters. Variants identified with a strand bias above 10 were removed from the final data set. Gene copy number variation was assessed by normalizing the sequencing depth of coverage for each gene in the sequenced genomes. Normalization was calculated by dividing the mean depth of coverage for each gene by the mean depth of coverage for the whole genome.

Insertion sequence (IS) elements were identified by searching against the ISfinder [48] database via the ISsaga [49] web server (available at http://issaga.biotoul.fr/issaga_index.php) using the *w*Mel reference genome. Identified IS queries that had greater than 80 % similarity (Table S3) were mapped to the short-read sequencing data for each sample using ISMapper [50] with default parameters to identify IS insertion sites. Insertion or deletion of IS elements identified as imprecise (*) or uncertain (?) were considered mapping artefacts.

A hybrid assembly was performed for all samples using all long-reads, and non-*Ae. aegypti* Illumina reads using Unicycler V0.4.7 [51] with default parameters. Long-reads were not pre-filtered to exclude *Ae. aegypti* reads, due to the lower accuracy of these reads being unsuitable for use with BioBloom Tools. However, Unicycler works to first assemble short-reads (pre-filtered) before scaffolding these assemblies together with the long-reads, meaning long-reads belonging to *Ae. aegypti* are essentially ignored. Genomes were trimmed and rotated to the *dnaA* gene, then confirmed closed in Unicycler. Single circularized genomes were assembled for seven samples from 2018. Gene synteny was assessed using Progressive Mauve software [52] (available at http://darlinglab.org/mauve/mauve.html), using standard parameters.

## Results

**Fig. 1.**
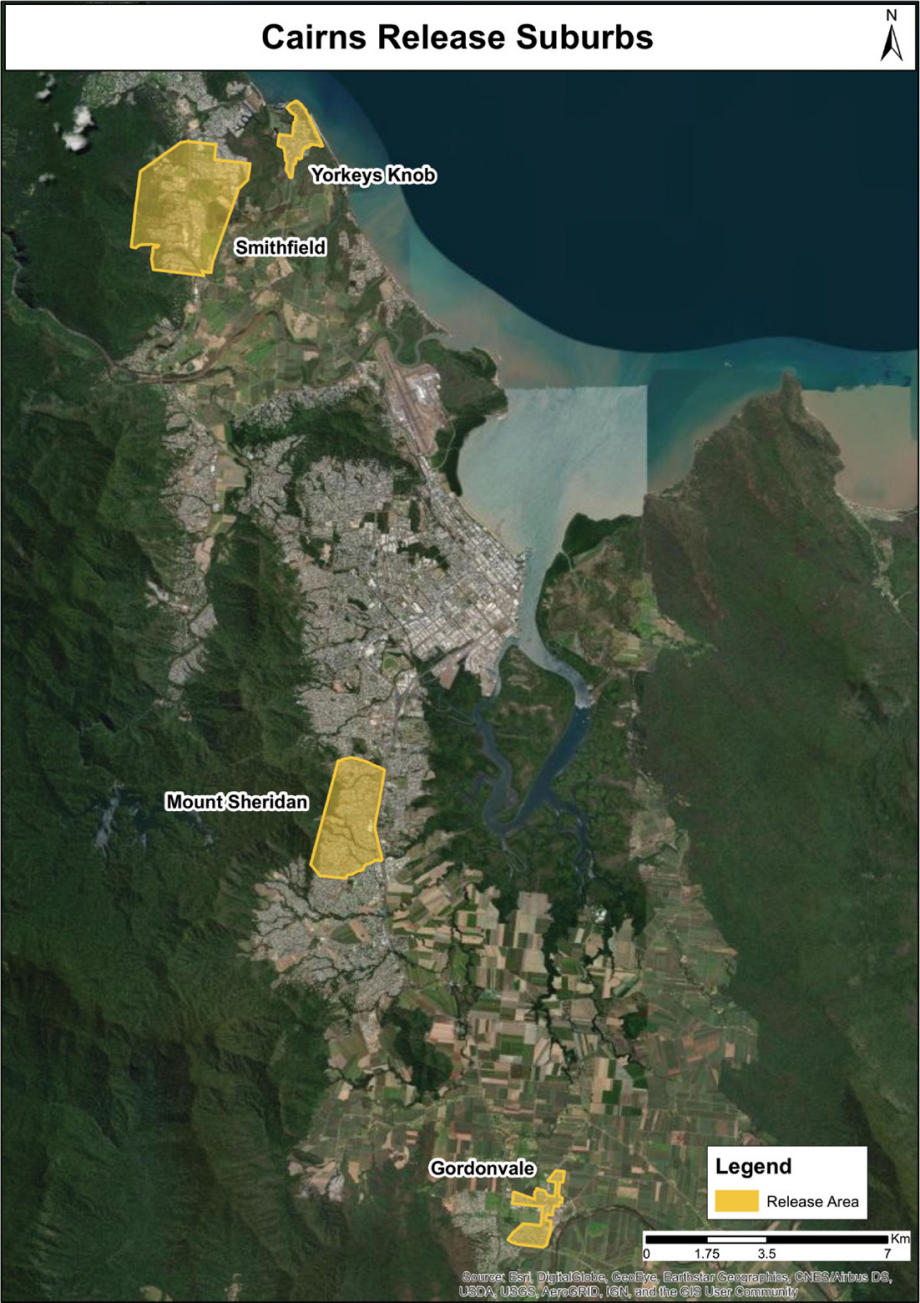
Locations in the greater Cairns area, North Queensland, where egg samples for this study were obtained. Releases occurred in 2011 in Yorkeys Knob and Gordonvale, and in 2017 in Smithfield and Mount Sheridan.

**Fig. 2.**
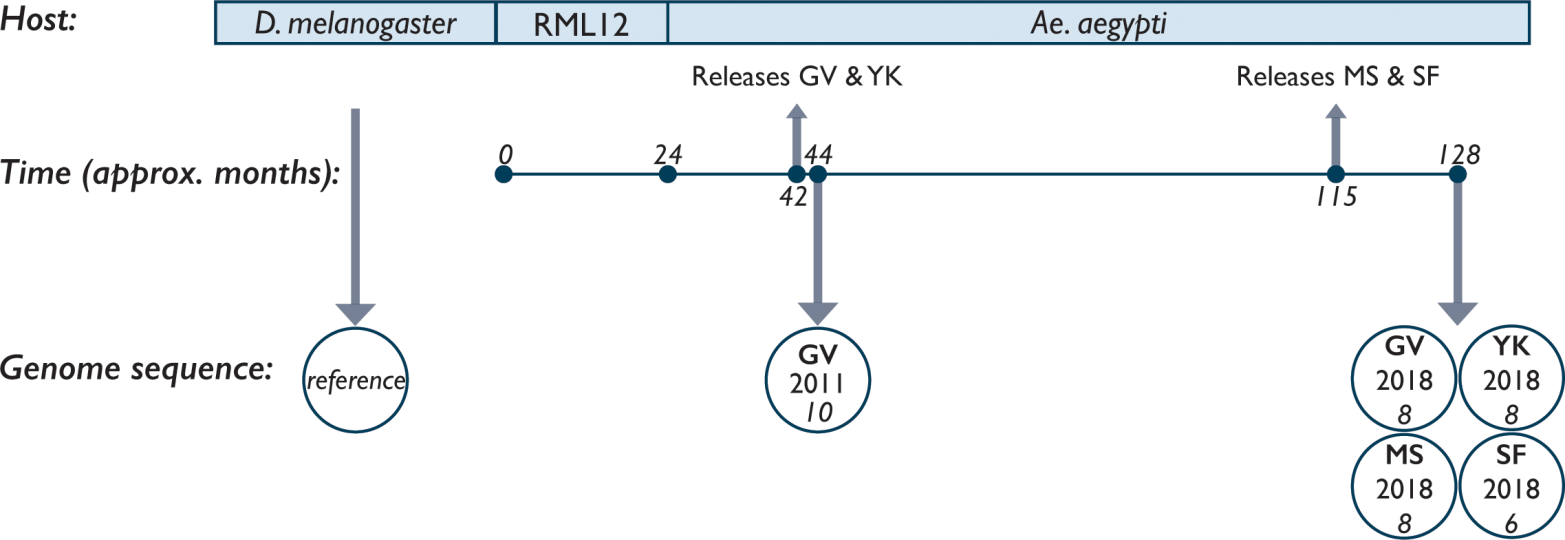
Timeline of the establishment and sampling of *w*Mel described in this study. The *Wolbachia* strain *w*Mel was purified from *D. melanogaster* and transinfected into the *Ae. albopictus*-derived cell line RML12. After approximately 24 months of serial passaging, the *w*Mel was transinfected into *Ae. aegypti* mosquitoes. Releases of *w*Mel-infected mosquitoes into the suburbs of Gordonvale (GV) and Yorkeys Knob (YK), and Mount Sheridan (MS) and Smithfield (SF), began approximately 42 and 115 months post-initial transfection, respectively. We sequenced the genomes of *w*Mel from mosquitoes at two timepoints, 2011 and 2018; the numbers of genomes sequenced from each suburb are indicated under the suburb initials and year.

**Fig. 3.**
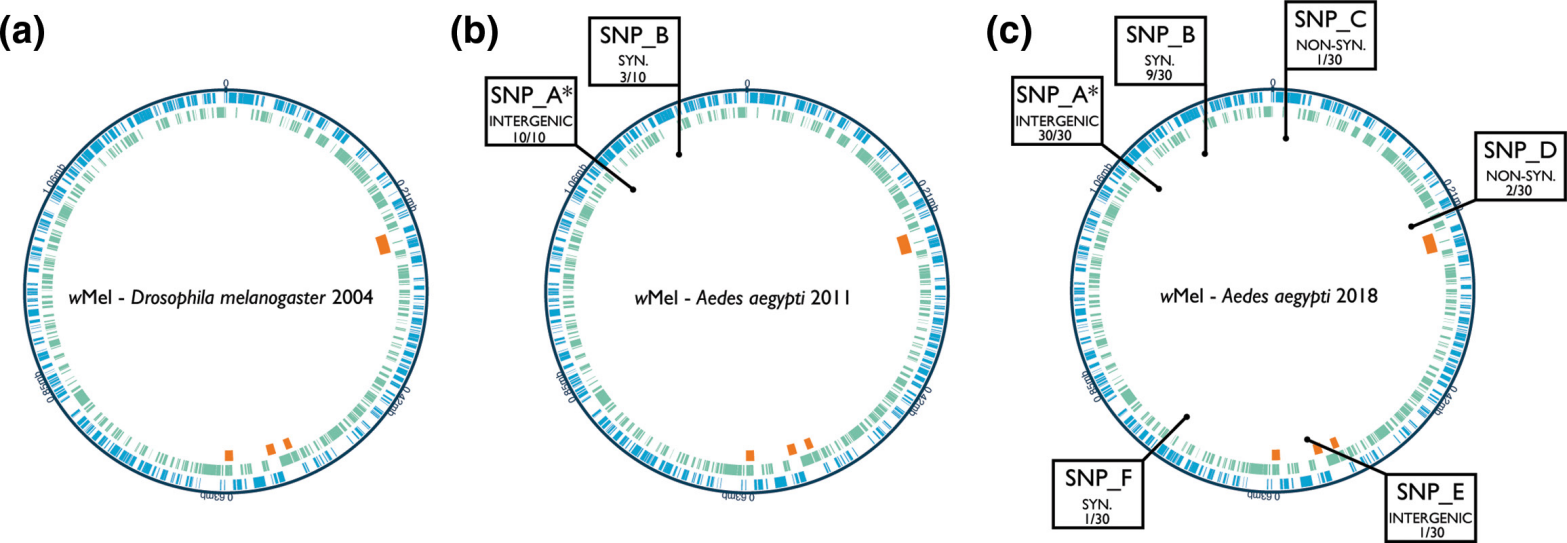
*w*Mel *Wolbachia* genome stability over time. (**a**) Progenitor *w*Mel strain sequenced from *D. melanogaster* in 2004. (**b**) *w*Mel strain in released *Ae. aegypti* sampled in 2011. (**c**) *w*Mel strain in released *Ae. aegypti* sampled in 2018. Circles correspond to the following: blue layer, forward strand genes; green layer, reverse strand genes; orange segments, phage regions – in clockwise order WO-A, pyocin-like, WO-B, WO-B. SNPs are designated letter labels (A–F) with mutation outcome listed as either intergenic, synonymous (SYN) or non-synonymous (NON-SYN), as well as number of samples in which the SNP was found. *SNP_A is an error in the reference sequence rather than a novel SNP since *w*Mel transfer to *Ae. aegypti*.

The first releases of *w*Mel-infected *Ae. aegypti* began in January 2011 in the Cairns, Australia, suburbs of Gordonvale and Yorkeys Knob (Fig. 1). In order to identify changes in the *w*Mel genome since its transinfection from *D. melanogaster* to *Ae. aegypti*, *w*Mel-infected larval samples collected from Gordonvale, Cairns, in 2011 were sequenced (Fig. 2). Analysis of *w*Mel *Wolbachia* genomes collected from Gordonvale ascertained the presence of two SNPs, compared to the previously described reference genome of *w*Mel (sequenced directly from its native host, *D. melanogaster*) (Fig. 3, Table 1). The first SNP, labelled SNP_A, was present in all 10 samples, and represents an intergenic T-A change at position 1097797 of the *w*Mel reference genome. The second SNP found in the 2011 samples, SNP_B, was present in 3 of 10 samples, and represents a synonymous T-C change in the hypothetical protein WD1228 at position 1174712. Seven indel events were also identified compared to the reference genome, each present in all 10 samples (Table 2). Short-read sequencing data was also used to assess IS movement within the genomes using the ISsaga and ISMapper programmes. No evidence of novel insertions or deletions of any IS elements were identified. Finally, gene copy number variation was assessed by normalizing the mean sequencing depth for each gene by the mean depth coverage of the entire genome. No distinct change in gene copy number was observed in any of the sequenced genomes (Fig. S1a). These data suggest low levels of *w*Mel genome polymorphism have occurred as a result of host transfer.

**Table 1.** SNPs present in *w*Mel *Wolbachia* from *Ae. aegypti* collected in 2011 and 2018 compared to the *w*Mel reference sequence from *D. melanogaster*

SNP	Position	Reference nucleotide	Alternate nucleotide	Change outcome	Gene	Ancestral codon	Derived codon	Ancestral amino acid	Derived amino acid	Gene product
SNP_A	1 097 797	T	A	Intergenic	–	–	–	–	–	–
SNP_B	1 174 712	T	C	Synonymous	WD_1228	CCT	CCC	P	P	Hypothetical protein
SNP_C	17 401	A	G	Non-synonymous	WD_0017	AGA	GGA	R	G	Translation elongation factor TU
SNP_D	229 585	C	A	Non-synonymous	WD_0244	CAG	CAT	Q	H	Hypothetical protein
SNP_E	587 862	A	G	Intergenic	–	–	–	–	–	–
SNP_F	754 656	G	A	Synonymous	WD_0780	TCG	TCA	S	S	DNA polymerase III, α subunit

SNP_A is an error in the reference sequence rather than a novel SNP since *w*Mel transfer to *Ae. aegypti*.

**Table 2.** Indel differences identified between the *w*Mel reference genome from *D. melanogaster* and the genomes sequenced in this study

Position	Reference nucleotide	Alternate nucleotide	ORF effect	Gene	Gene product
1 006 081	GT	GTT	Frameshift	WD_1044	Hypothetical protein
1 020 475	CTTTT	CTTTTT	Intergenic	–	–
1 094 458	GTT	GTTT	Frameshift	WD_1143	ispD/ispF bifunctional enzyme
1 103 468	ATTTT	ATTT	Frameshift	WD_1155	Hypothetical protein
1 161 850	GTTTTT	GTTTTTT	Intergenic	–	–
1 163 170	GC	G	Frameshift	WD_1215	Hypothetical protein
1 177 853	ACC	AC	Frameshift	WD_1231	Hypothetical protein

These indels are errors in the reference sequence rather than novel indels since *w*Mel transfer to *Ae. aegypti*.

Since 2011, releases of *w*Mel-infected *Ae. aegypti* populations have occurred throughout the regional cities of Cairns, including Mount Sheridan and Smithfield, which were part of releases completed in 2017 (Figs 1 and 2). Longitudinal monitoring of *Ae. aegypti w*Mel infection frequency indicates *Wolbachia* introgression has remained stable throughout these areas since establishment [26]. *w*Mel-infected mosquitoes were collected in 2018 from Gordonvale and Yorkeys Knob in order to ascertain genomic changes accrued in the field, in the 86 months since the end of releases. Alongside this, *w*Mel-infected mosquitoes from Mount Sheridan and Smithfield were also collected in 2018. These samples represented a shorter time in the field of 13 and 10 months, respectively, but may have been more diverse by virtue of genetic drift due to the *w*Mel mosquito line having been maintained as a smaller laboratory population until release (Fig. 2).

All samples from 2018 were sequenced and analysed in the same manner as those from 2011. Again, SNP_A was identified in all samples from 2018 (Fig. 3, Table 1). Interestingly, SNP_B was observed in 9/30 samples, a similar proportion as in 2011, and was located across all four suburbs (13–50 %, depending on suburb). A further four SNPs were identified across the 2018 samples, but were only present in one or two samples. SNP_C, E and F were each identified in one sample from Smithfield, Yorkeys Knob and Mount Sheridan, respectively, and cause non-synonymous, intergenic and synonymous changes. SNP_D, which causes a non-synonymous change in hypothetical protein WD_0244, was observed in two samples, one from Mount Sheridan and one from Yorkeys Knob. As WD_0244 represents a small hypothetical protein, one concern was that it has been misannotated. However, a study looking at *w*Mel gene expression across *D. melanogaster* developmental stages showed WD_0244 to be expressed across multiple life stages, supporting its current annotation [53]. Of these novel four SNPs, no greater proportion was observed in the older sites, Gordonvale and Yorkeys Knob, compared to the more recent sites, Smithfield and Mount Sheridan (Table S4). Each of the seven indel differences identified in the 2011 samples were again present in all of the 2018 samples (Table 2). As with 2011 samples, no evidence of novel insertions or deletions of any IS elements were identified. Again, no distinct change in gene copy number variation was observed in any of the sequenced genomes (Fig. S1b–e). These data suggest field establishment is having little effect on the *w*Mel genome.

While the goal of our study was to identify SNPs that could have a potential impact on *w*Mel-induced phenotypes, we also identified low-frequency variants within individual samples as this would provide an indication of the mutational input on which selection and drift can act. LoFreq was used to identify low-frequency SNPs from the short-read sequencing data for all 2018 samples (File S1). We identified a total of 281 variants across all samples in addition to our previously identified SNPs. Of the variants identified, 74 % had an allele frequency <10 %, and 98.6 % had an allele frequency <20 %. Frequency variation was also assessed for the six fixed SNPs identified in this study across all genomes sequenced (Fig. S2). SNP_B, which was found to be fixed in 12 of 40 genomes sequenced, was found at lower frequencies in a further nine genomes from the 2018 collections. SNP_E and SNP_F, which were each found to be fixed in 1 of 40 sequenced genomes, were found at lower frequencies in one additional genome each from the 2018 collections. As all samples from 2018 were generated by pooling material, this represents the frequency of the pool, rather than heteroplasmy within individual mosquitoes.

Long-read sequencing was undertaken on the 2018 samples collected from Gordonvale using the Oxford Nanopore platform to allow for the identification of large structural rearrangements. The Unicycler program was used to perform hybrid *de novo* genome assemblies using both the short- and long-read data. Seven 2018 Gordonvale genomes were able to be completely resolved, and genomes closed. The Mauve program was used to identify changes to gene order or genome rearrangement in the seven resolved genomes from Gordonvale in comparison to the *D. melanogaster w*Mel reference genome. No changes were observed in any of the genomes compared to the reference genome (Fig. S3). Each genome aligned in full to every other genome, creating single homologous blocks that indicate the genomes are internally free from genomic rearrangement.

## Discussion

The *w*Mel strain of *Wolbachia* has been introgressed into field populations of *Ae. aegypti* since 2011 as an intervention for reducing the prevalence of arboviruses. Observational data from across Australia show a reduction in dengue incidence since the introgression of *Wolbachia* into Cairns and Townsville [26, 27]. This intervention has proven effective due to the maintenance of viral inhibition and CI caused by *w*Mel infection after its artificial transfer from its native host, *D. melanogaster*, to *Ae. aegypti* [13]. However, previous transinfections using a closely related *Wolbachia* strain, *w*MelPop, resulted in substantial changes to its genome after host transfer. Therefore, we hypothesized that this host transfer, along with the introgression of *w*Mel into wild *Ae. aegypti* populations with much larger population sizes than lab-reared colonies, may have caused substantial genomic changes. Our results, however, have shown a remarkably stable genome.

A total of six SNPs were identified across the 2011 and 2018 field samples. Only one SNP (SNP_A, T → A) was present in all 2011 and 2018 samples. However, when queried further, this position was found to contain an ‘A’ in 100 % of genome sequences in three separate studies of *w*Mel genomes from *D. melanogaster* [38, 39, 54]. Therefore, we obtained the Sanger sequencing trace data from the original *w*Mel reference genome. The Sanger traces indicated a miscall at this position, with trace data indicating a clear ‘A’ base, rather than the called ‘T’ base (Fig. S4a). In combination, this data provides clear evidence that SNP_A is instead an error in the reference genome, rather than a substitution that has occurred since transinfection of *w*Mel into *Ae. aegypti*. The seven indel events identified in this study were also present in all 2011 and 2018 sequences. Each of these was again examined in the Sanger sequencing trace data from the original *w*Mel reference genome. For six out of seven of these indel events (positions 1 006 081, 1 020 475, 1 094 458, 1 161 850, 1163 170 and 1 177 853) there is strong evidence they are errors in the reference sequence (Fig. S4b–d, f–h). For the other indel at position 1 103 468, sequencing traces do not clearly indicate the number of Ts present at the position (Fig. S4e). However, another published *w*Mel genome sequence from *D. melanogaster* reports only three Ts at this position, rather than the four present in the reference genome, supporting this indel is also likely an error [55]. Given these results, it is clear these indel events are again errors in the reference sequence, rather than mutations that have occurred since transinfection of *w*Mel into *Ae. aegypti*.

The only other SNP identified in both 2011 and 2018 (SNP_B) represents a synonymous change in a hypothetical protein. As it was identified at a similar frequency at both time points, and present in all sampled suburbs, it is likely this change was present in the mosquito release colony and is providing no strong evolutionary benefit or disadvantage. The remaining four SNPs identified in this study were present in only 2018, and in one or two samples. The SNP found in two samples, SNP_D, was observed in two suburbs separated from one another by approximately 17 km (Yorkeys Knob and Mount Sheridan), making migration of mosquitoes between them unlikely. Unintentional human transportation of mosquitoes carrying this SNP may have occurred, however, or alternatively this SNP may have arisen independently, twice, potentially indicating this to be an adaptation to field inhabitancy. No evidence of novel insertions or deletions of any IS elements were identified in any of the sequences generated in this study. However, a higher depth of sequencing would increase possible avenues to explore this, as previous studies show the confidence in IS annotation increases with sequencing depth for the type of analysis performed in this study [50].

Our 2018 field sampling included mosquitoes from sites established in 2011, as well as 2017. Interestingly, the older release sites, Gordonvale and Yorkeys Knob, exhibited no greater abundance of genomic changes than the later release sites, Smithfield and Mount Sheridan, although the total number of polymorphisms is low. The two later release sites studied, Smithfield and Mount Sheridan, share no common polymorphisms despite these releases occurring around the same time and from the same colony stock. This suggests polymorphisms have not accumulated in the lab colony stock since the initial field releases in 2011. Importantly, no changes were identified in the genes known to be responsible for the CI phenotype, *cifA* and *cifB* [32–34, 56], indicating a stability of this desired trait. As the mechanism of *Wolbachia *-induced viral inhibition is yet unknown, no such conclusion can be made for this phenotype. However, the general stability of the genome predicts the trait is not at high risk of being lost due to changes in the *Wolbachia* genome. Consistent with this, a stable virus blocking phenotype was reported in field-derived *Ae. aegypti*, sampled from Cairns, Australia, 12 months post-release in 2012 [25].

Huang *et al*. [29] recently reported the sequencing of *w*Mel from colony material, as well as *w*Mel-infected mosquitoes collected 8 years post-release in Australia. While the general observations of *w*Mel genome stability are observed in both studies, our sampling and sequencing methods have allowed for a more in-depth analysis of *w*Mel genome evolution. Our baseline sampling comprised 10 individual larvae collected in 2011, whereas Huang *et al*. used a single pool of 400 mosquitoes from material that was collected from the field in 2013 and reared in the laboratory until its sequencing in 2019. This allowed us to identify the polymorphism labelled SNP_B in both 2011 as well 2018 samples, whereas Huang *et al*. 2020 only identified this SNP in 2019. Our use of 2011 material allowed us to confirm that SNP_B was present in *w*Mel release material and did not originate in the field. Our study also found three additional SNPs in 2018 sampling (SNP_C, E and F) compared to that of Huang *et al*. study in 2019. However, only two of the suburbs assessed overlapped in the two studies. Thus, these SNPs may be unique to the suburbs sampled in this study as each were present at low frequency. Finally, our study also included the use of long-read sequencing, allowing us to fully resolve *w*Mel genomes and conclude that no genome rearrangements have occurred since transfer to *Ae. aegypti* or release in the field.

This low level of variation coupled with the lack of IS movement and genomic rearrangements in the *w*Mel genome is somewhat surprising given the occurrence of host transfer and introduction to a novel environment. A number of genomic changes were observed in the closely related strain of *Wolbachia, w*MelPop, after it underwent transinfection into a novel host. This strain, however, was transinfected into an additional *Aedes albopictus-*derived cell line, Aa23, which may have provided additional stresses leading to the genomic changes [30]. When low-frequency variation was assessed in this study, a large number of variants were identified; however, the majority of variants were at a low frequency. This suggests there is mutational input upon which selection can act; however, these are not yet fixing in the population. Similar findings to ours have been reported for the *Wolbachia* strain *w*Cer2, which displayed stability of genome content and synteny, and low levels of sequence polymorphism in multiple novel hosts [57]. Alongside this, studies of the aphid endosymbiont *Buchnera* across different aphid species have reported low levels of DNA polymorphism, which was posited to be more likely shaped by symbiosis effects of aphids and *Buchnera* (bottleneck effects during maternal transmission and population fluctuations) rather than by features incidental to different aphid species [36, 58, 59].

The low level of variation observed here could be due to multiple explanations. It is possible that the novel host of *w*Mel, *Ae. aegypti*, does not differ enough in terms of population dynamics and vertical endosymbiont transfer from its predecessor, *D. melanogaster*, to drive selective pressures on the *w*Mel genome. Additionally, it is possible that the observed low-frequency mutations are under strong purifying selection, or that population bottlenecks associated with maternal transmission are limiting their inheritance. Furthermore, considering the relatively short time *w*Mel had been established in the field when sequenced (~86 months), coupled with the reportedly low estimated mutation rate of *w*Mel (6.8×10^−10^ substitutions per site per *D. melanogaster* generation [38, 39]), more time may be required to amass greater differences. Using the mutation rate of 6.8×10^−10^ substitutions per site per generation and the estimated number of 104 generations (assuming one generation per month) that *w*Mel has been in *Ae. aegypti* (Fig. 2), we would expect to observe 0.0897 base substitutions across each genome. This estimation is consistent with what we have observed in this study. It is also known that the *w*Mel genome has a number of partially intact DNA repair genes [28, 60], possibly limiting the number of polymorphisms that are able to fix in the germline.

The multi-faceted sequencing and analysis techniques used in this study have allowed for the establishment of complete *w*Mel *Wolbachia* sequences and demonstrated a remarkably stable genome in terms of sequence, gene content, and structure. These results provide some of the first data regarding the genome stability of *w*Mel. This, combined with recent field entomology data showing the stability of *w*Mel-infection prevalence, gives reassurance on the potential for *w*Mel to deliver long-term public-health impacts. Future studies should continue to monitor both the genomic evolution of the *w*Mel genome, as well as the phenotypic features of viral inhibition and CI.

## Supplementary Data

Supplementary material 1

Supplementary material 2
