# Supplementary material for: wMel Wolbachia genome remains stable after 7 years in Australian Aedes aegypti field populations
Source: Microb Genom. 2021 Sep 1;7(9):000641. doi: 10.1099/mgen.0.000641 (PMC8715424; doi:10.1099/mgen.0.000641)
Supplement: Supplementary material 1 [file mgen-7-0641-s001.pdf]

Supporting Information

S1 Table. Dates of *Wolbachia* release and collection, with total number of genomes sequenced per suburb.

SUBURB	RELEASE DATE	COLLECTION DATE	TOTAL SAMPLES SEQUENCED
**GORDONVALE**	Jan 2011	Mar-Apr 2011	10
		Mar 2018	8
**YORKEYS KNOB**	Jan 2011	Mar-May 2018	8
**MOUNT SHERIDAN**	Feb 2017	Mar-May 2018	8
**SMITHFIELD**	May 2017	Mar-May 2018	6

S2 Table. Sample information. Information regarding sample name, NCBI BioSample accession number, SRA number(s), Genome accession number, location and time of collection, sequencing platform(s) used, and identified features. SNP_A and indels were identified in every genome in this study. Therefore, these are not noted in the table.

SAMPLE NAME	ACCESSION NUMBER	SRA	GENOME ACCESSSION NUMBER	COLLECTION SOURCE	SEQUENCING PLATFORM	IDENTIFIED FEATURES
**GV_2011_1**	SAMN17611319	SRR13566486	N/A	Gordonvale 2011	NovaSeq S1 300 bp	
**GV_2011_2**	SAMN17611320	SRR13566485	N/A	Gordonvale 2011	NovaSeq S1 300 bp	
**GV_2011_3**	SAMN17611321	SRR13566474	N/A	Gordonvale 2011	NovaSeq S1 300 bp	SNP_B
**GV_2011_4**	SAMN17611322	SRR13566463	N/A	Gordonvale 2011	NovaSeq S1 300 bp	
**GV_2011_5**	SAMN17611323	SRR13566452	N/A	Gordonvale 2011	NovaSeq S1 300 bp	
**GV_2011_6**	SAMN17611324	SRR13566451	N/A	Gordonvale 2011	NovaSeq S1 300 bp	SNP_B
**GV_2011_7**	SAMN17611325	SRR13566450	N/A	Gordonvale 2011	NovaSeq S1 300 bp	
**GV_2011_8**	SAMN17611326	SRR13566449	N/A	Gordonvale 2011	NovaSeq S1 300 bp	
**GV_2011_9**	SAMN17611327	SRR13566448	N/A	Gordonvale 2011	NovaSeq S1 300 bp	SNP_B
**GV_2011_10**	SAMN17611328	SRR13566447	N/A	Gordonvale 2011	NovaSeq S1 300 bp	
**GV_2018_1**	SAMN17611329	SRR13566484; SRR13566561	CP072672	Gordonvale 2018	MiniSeq High output 300 bp; ONT MinION	
**GV_2018_2**	SAMN17611330	SRR13566483; SRR13566560	CP072671	Gordonvale 2018	MiniSeq High output 300 bp; ONT MinION	
**GV_2018_3**	SAMN17611331	SRR13566482; SRR13566559	CP072670	Gordonvale 2018	MiniSeq High output 300 bp; ONT MinION	
**GV_2018_4**	SAMN17611332	SRR13566481; SRR13566558	CP072669	Gordonvale 2018	MiniSeq High output 300 bp; ONT MinION	
**GV_2018_5**	SAMN17611333	SRR13566480; SRR13566557	CP072668	Gordonvale 2018	MiniSeq High output 300 bp; ONT MinION	SNP_B

**GV_2018_6**	SAMN17611334	SRR13566479; SRR13566556	CP072667	Gordonvale 2018	MiniSeq High output 300 bp; ONT MinION	
**GV_2018_7**	SAMN17611335	SRR13566478; SRR13566555	CP072666	Gordonvale 2018	MiniSeq High output 300 bp; ONT MinION	
**GV_2018_8**	SAMN17611336	SRR13566477; SRR13566554	N/A	Gordonvale 2018	MiniSeq High output 300 bp; ONT MinION	
**MS_2018_1**	SAMN17611345	SRR13566467	N/A	Mount Sheridan 2018	NextSeq Mid output 300 bp	
**MS_2018_2**	SAMN17611346	SRR13566466	N/A	Mount Sheridan 2018	NextSeq Mid output 300 bp	
**MS_2018_3**	SAMN17611347	SRR13566465	N/A	Mount Sheridan 2018	NextSeq Mid output 300 bp	SNP_B
**MS_2018_4**	SAMN17611348	SRR13566464	N/A	Mount Sheridan 2018	NextSeq Mid output 300 bp	SNP_D
**MS_2018_5**	SAMN17611349	SRR13566462	N/A	Mount Sheridan 2018	NextSeq Mid output 300 bp	SNP_B
**MS_2018_6**	SAMN17611350	SRR13566461	N/A	Mount Sheridan 2018	NextSeq Mid output 300 bp	SNP_B
**MS_2018_7**	SAMN17611351	SRR13566460	N/A	Mount Sheridan 2018	NextSeq Mid output 300 bp	SNP_F
**MS_2018_8**	SAMN17611352	SRR13566459	N/A	Mount Sheridan 2018	NextSeq Mid output 300 bp	
**SF_2018_1**	SAMN17611353	SRR13566458	N/A	Smithfield 2018	NextSeq Mid output 300 bp	SNP_B
**SF_2018_2**	SAMN17611354	SRR13566457	N/A	Smithfield 2018	NextSeq Mid output 300 bp	SNP_B
**SF_2018_3**	SAMN17611355	SRR13566456	N/A	Smithfield 2018	NextSeq Mid output 300 bp	
**SF_2018_4**	SAMN17611356	SRR13566455	N/A	Smithfield 2018	NextSeq Mid output 300 bp	SNP_B
**SF_2018_5**	SAMN17611357	SRR13566454	N/A	Smithfield 2018	NextSeq Mid output 300 bp	SNP_C
**SF_2018_6**	SAMN17611358	SRR13566453	N/A	Smithfield 2018	NextSeq Mid output 300 bp	
**YK_2018_1**	SAMN17611337	SRR13566476	N/A	Yorkeys Knob 2018	NextSeq Mid output 300 bp	SNP_B
**YK_2018_2**	SAMN17611338	SRR13566475	N/A	Yorkeys Knob 2018	NextSeq Mid output 300 bp	
**YK_2018_3**	SAMN17611339	SRR13566473	N/A	Yorkeys Knob 2018	NextSeq Mid output 300 bp	
**YK_2018_4**	SAMN17611340	SRR13566472	N/A	Yorkeys Knob 2018	NextSeq Mid output 300 bp	SNP_B;SNP_D
**YK_2018_5**	SAMN17611341	SRR13566471	N/A	Yorkeys Knob 2018	NextSeq Mid output 300 bp	SNP_E
**YK_2018_6**	SAMN17611342	SRR13566470	N/A	Yorkeys Knob 2018	NextSeq Mid output 300 bp	
**YK_2018_7**	SAMN17611343	SRR13566469	N/A	Yorkeys Knob 2018	NextSeq Mid output 300 bp	
**YK_2018_8**	SAMN17611344	SRR13566468	N/A	Yorkeys Knob 2018	NextSeq Mid output 300 bp	

S3 Table. List of IS queries identified to have greater than 80% similarity to the *w*Mel reference genome.

IS ELEMENT	FAMILY	GROUP
**ISCAA8**	IS5	IS903
**ISWEN1**	IS4	IS231
**ISWEN2**	IS110	
**ISWEN3**	IS66	ISBst12
**ISWOSP2**	IS4	IS50
**ISWPI1**	IS5	IS1031
**ISWPI11**	IS630	
**ISWPI12**	IS110	
**ISWPI14**	IS110	
**ISWPI15**	IS256	
**ISWPI18**	IS4	IS4
**ISWPI4**	IS481	

S4 Table. Suburb locations of SNPs identified in 2018 samples.

SNP	FREQUENCY (OF 30)	LOCATIONS
**SNP_A**	30	Gordonvale (8/8), Yorkeys Knob (8/8), Smithfield (6/6), Mount Sheridan (8/8)
**SNP_B**	9	Gordonvale (1/8), Yorkeys Knob (2/8), Smithfield (3/6), Mount Sheridan (3/8)
**SNP_C**	1	Smithfield (1/6)
**SNP_D**	2	Yorkeys Knob (1/8), Mount Sheridan (1/8)
**SNP_E**	1	Yorkeys Knob (1/8)
**SNP_F**	1	Mount Sheridan (1/8)

*SNP_A, SNP_B, and SNP_D were identified in the Huang et al., 2020 study also, identified by genome positions 1097797, 1174712, and 229585.

A)

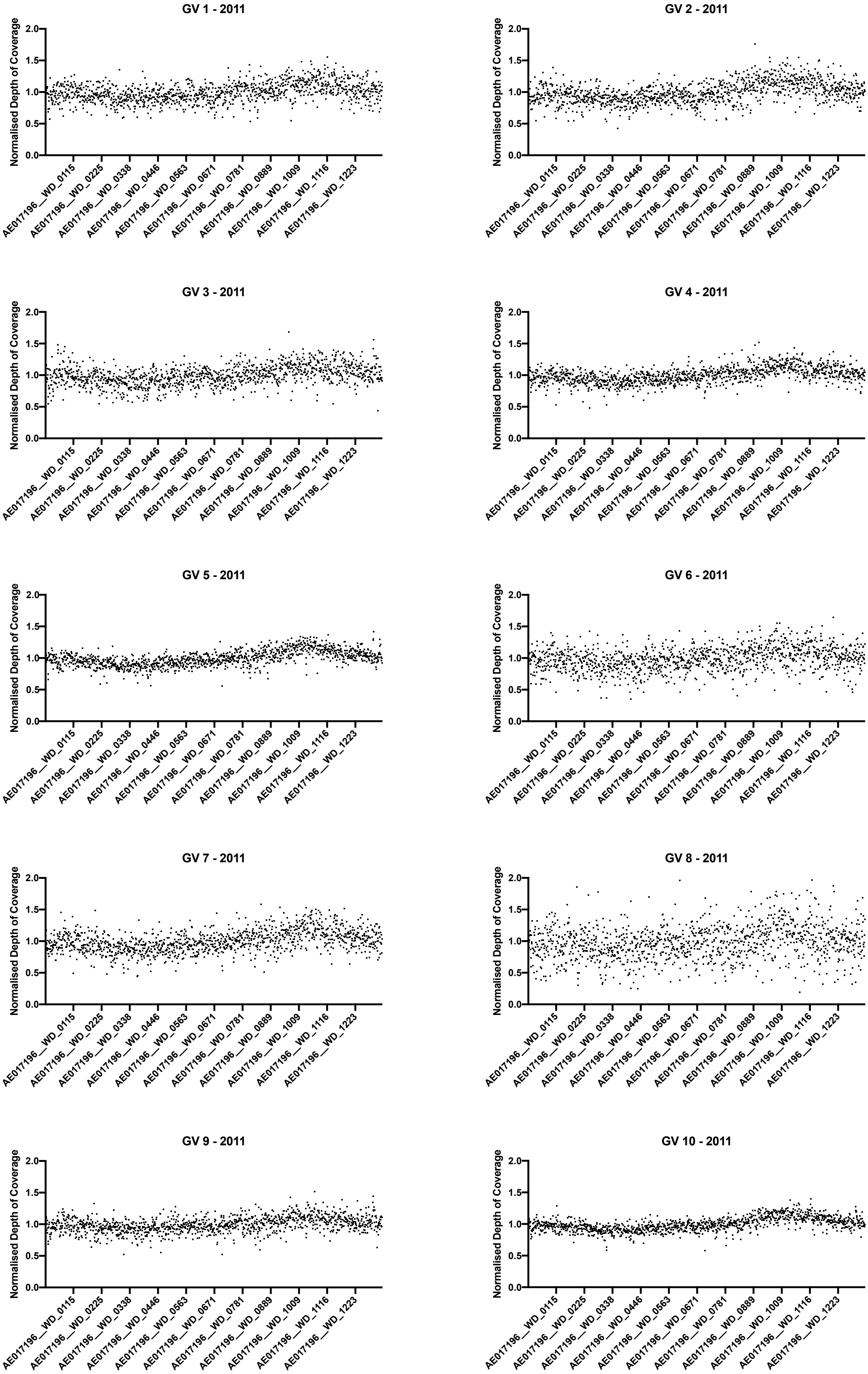
GV 1 - 2011
GV 2 - 2011
GV 3 - 2011
GV 4 - 2011
GV 5 - 2011
GV 6 - 2011
GV 7 - 2011
GV 8 - 2011
GV 9 - 2011
GV 10 - 2011
Normalised Depth of Coverage
0.0
0.5
1.0
1.5
2.0
AE017196__WD_0115
AE017196__WD_0225
AE017196__WD_0338
AE017196__WD_0446
AE017196__WD_0563
AE017196__WD_0671
AE017196__WD_0781
AE017196__WD_0889
AE017196__WD_1009
AE017196__WD_1116
AE017196__WD_1223

B)

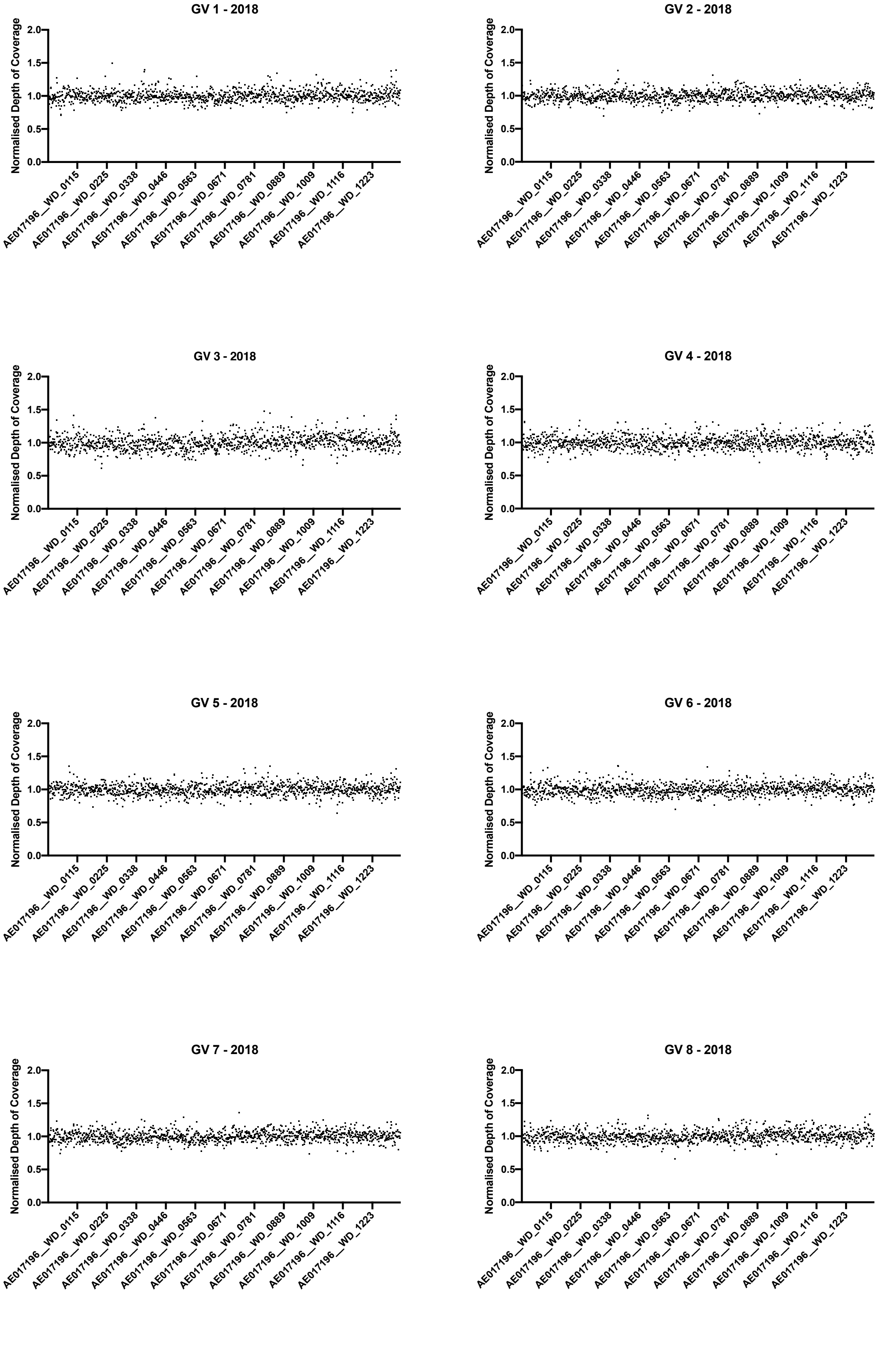
GV 1 - 2018
GV 2 - 2018
GV 3 - 2018
GV 4 - 2018
GV 5 - 2018
GV 6 - 2018
GV 7 - 2018
GV 8 - 2018
Normalised Depth of Coverage
0.0
0.5
1.0
1.5
2.0
AE017196__WD_0115
AE017196__WD_0225
AE017196__WD_0338
AE017196__WD_0446
AE017196__WD_0563
AE017196__WD_0671
AE017196__WD_0781
AE017196__WD_0889
AE017196__WD_1009
AE017196__WD_1116
AE017196__WD_1223

C)

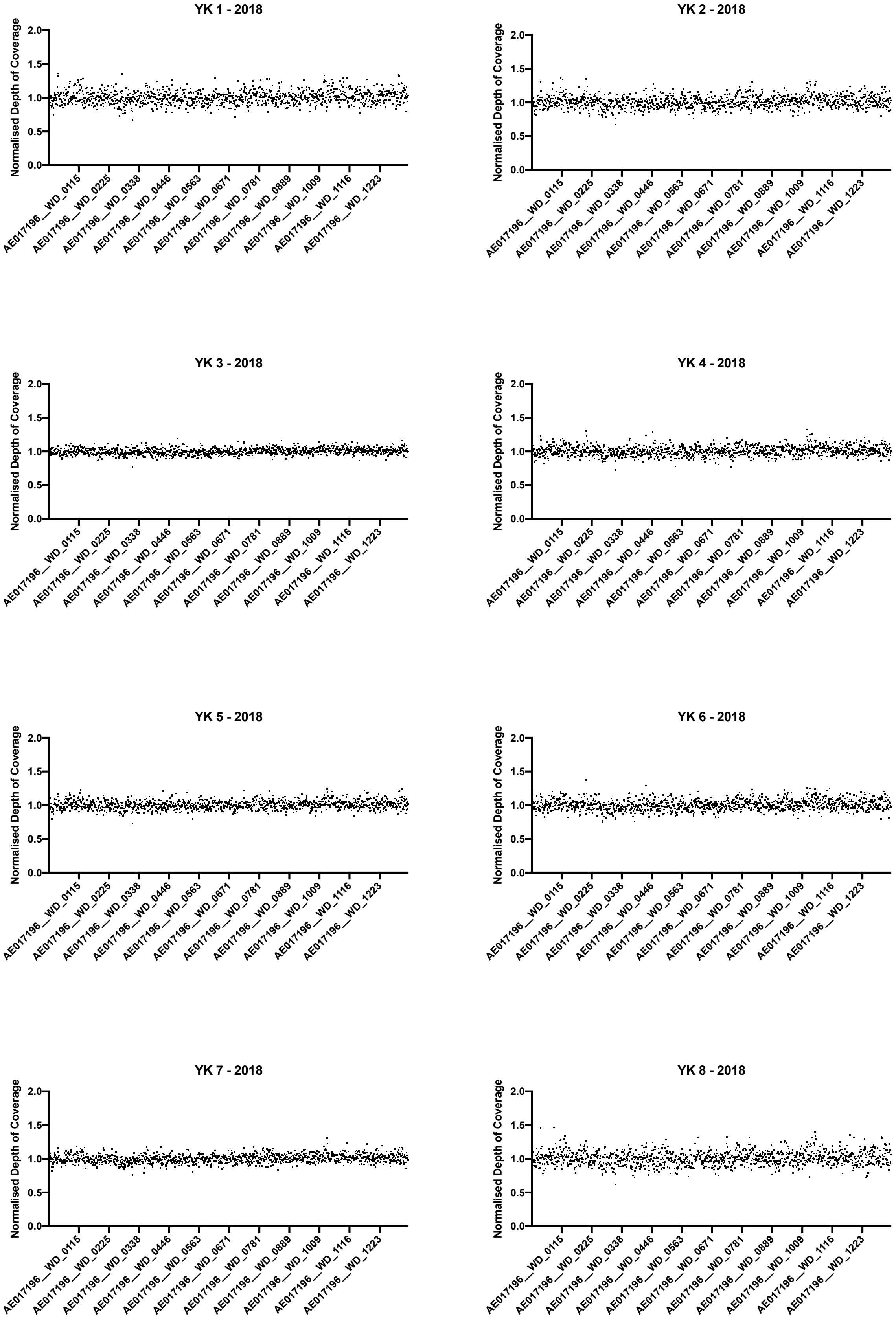
YK 1 - 2018
YK 2 - 2018
YK 3 - 2018
YK 4 - 2018
YK 5 - 2018
YK 6 - 2018
YK 7 - 2018
YK 8 - 2018
Normalised Depth of Coverage
0.0
0.5
1.0
1.5
2.0
AE017196__WD_0115
AE017196__WD_0225
AE017196__WD_0338
AE017196__WD_0446
AE017196__WD_0563
AE017196__WD_0671
AE017196__WD_0781
AE017196__WD_0889
AE017196__WD_1009
AE017196__WD_1116
AE017196__WD_1223

D)

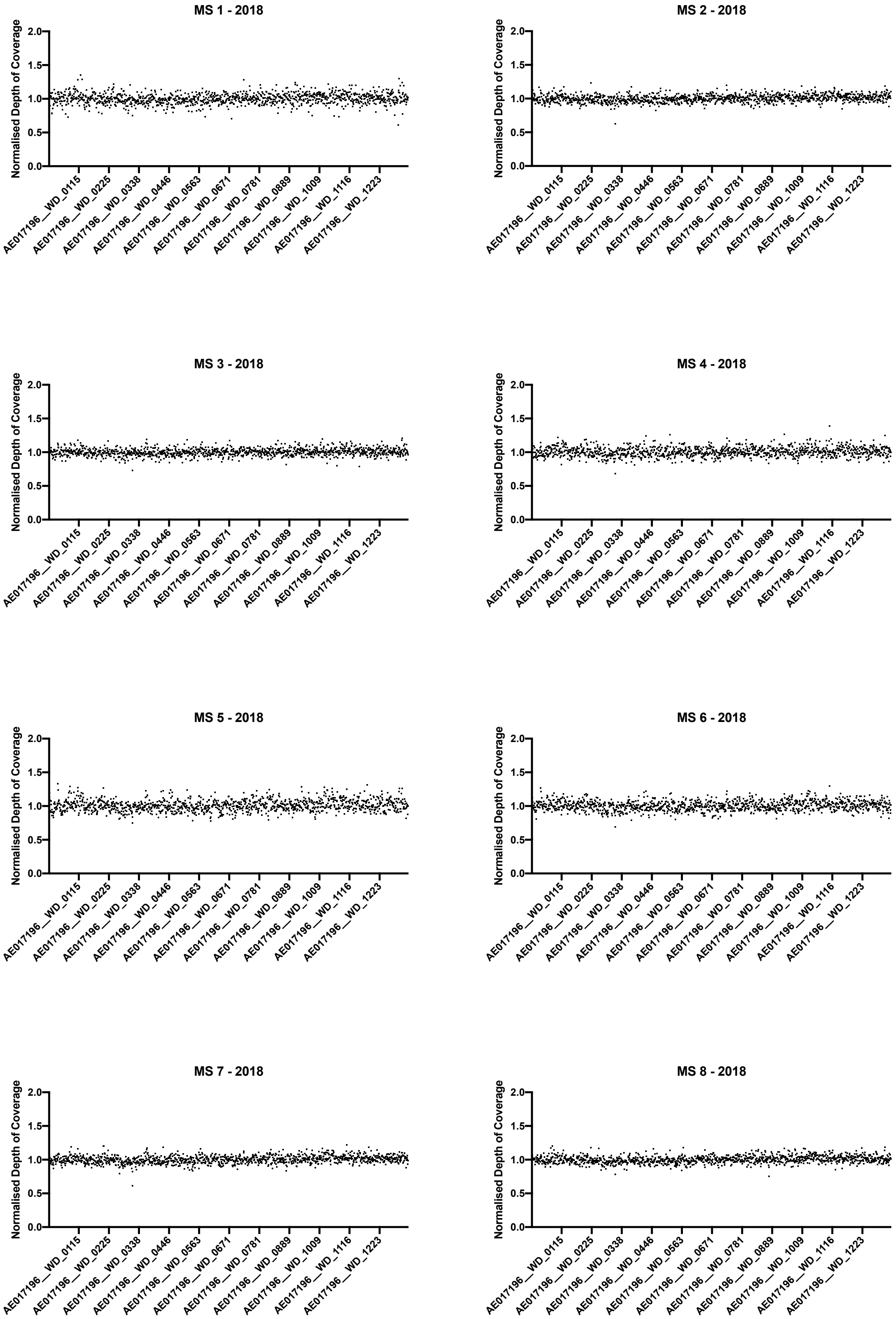
MS 1 - 2018
MS 2 - 2018
MS 3 - 2018
MS 4 - 2018
MS 5 - 2018
MS 6 - 2018
MS 7 - 2018
MS 8 - 2018
Normalised Depth of Coverage
0.0
0.5
1.0
1.5
2.0
AE017196__WD_0115
AE017196__WD_0225
AE017196__WD_0338
AE017196__WD_0446
AE017196__WD_0563
AE017196__WD_0671
AE017196__WD_0781
AE017196__WD_0889
AE017196__WD_1009
AE017196__WD_1116
AE017196__WD_1223

E)

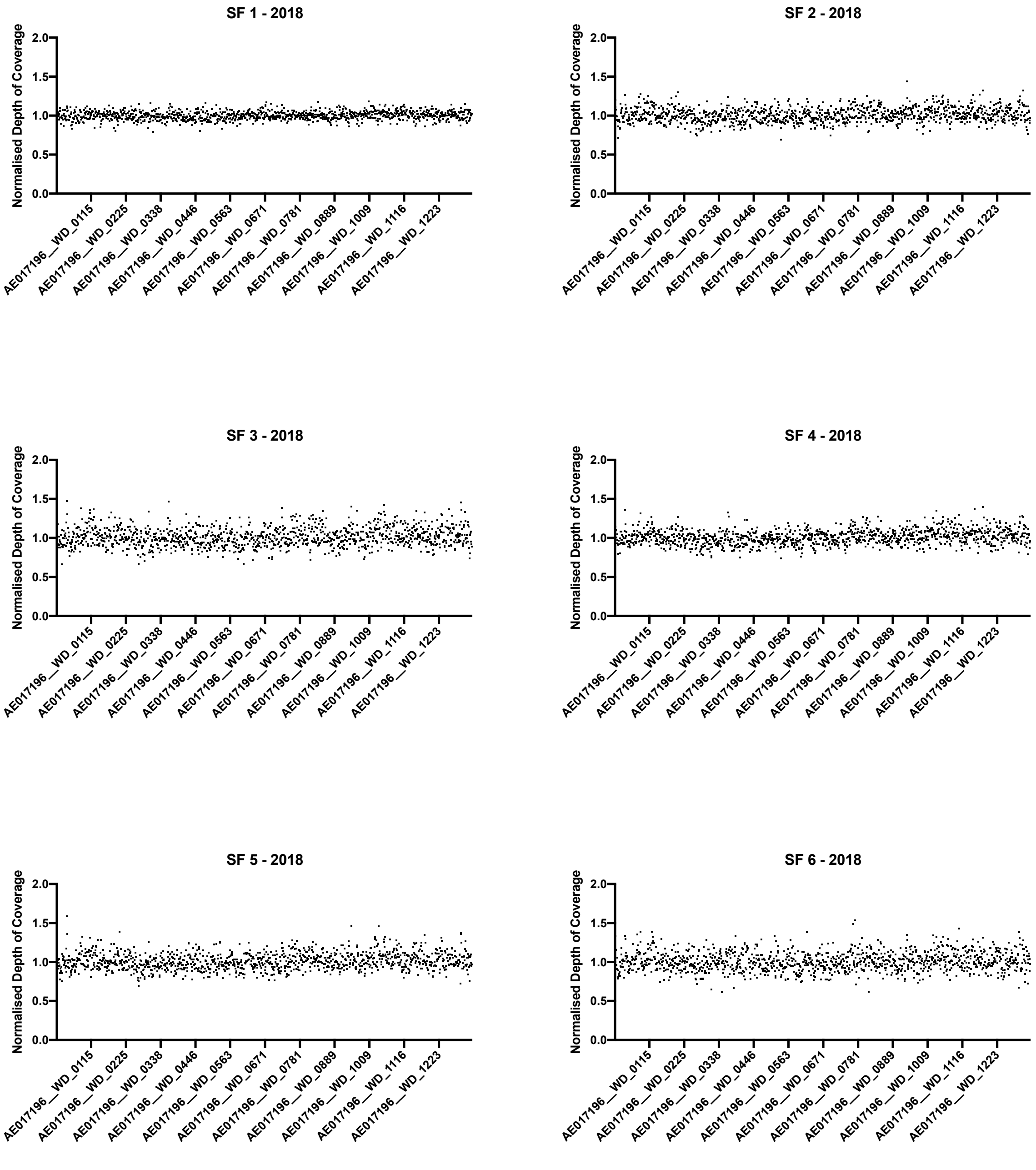

S1 Fig. Normalised sequencing depth of coverage. The distribution of coverage is plotted for all genes in each sequenced genome from A) Gordonvale – 2011, B) Gordonvale – 2018, C) Yorkeys Knob – 2018, D) Mount Sheridan – 2018, E) Smithfield – 2018. Each data point represents the normalised depth of coverage for that gene. Normalisation was calculated by dividing the depth of coverage for each gene by the mean depth of coverage for the whole genome.

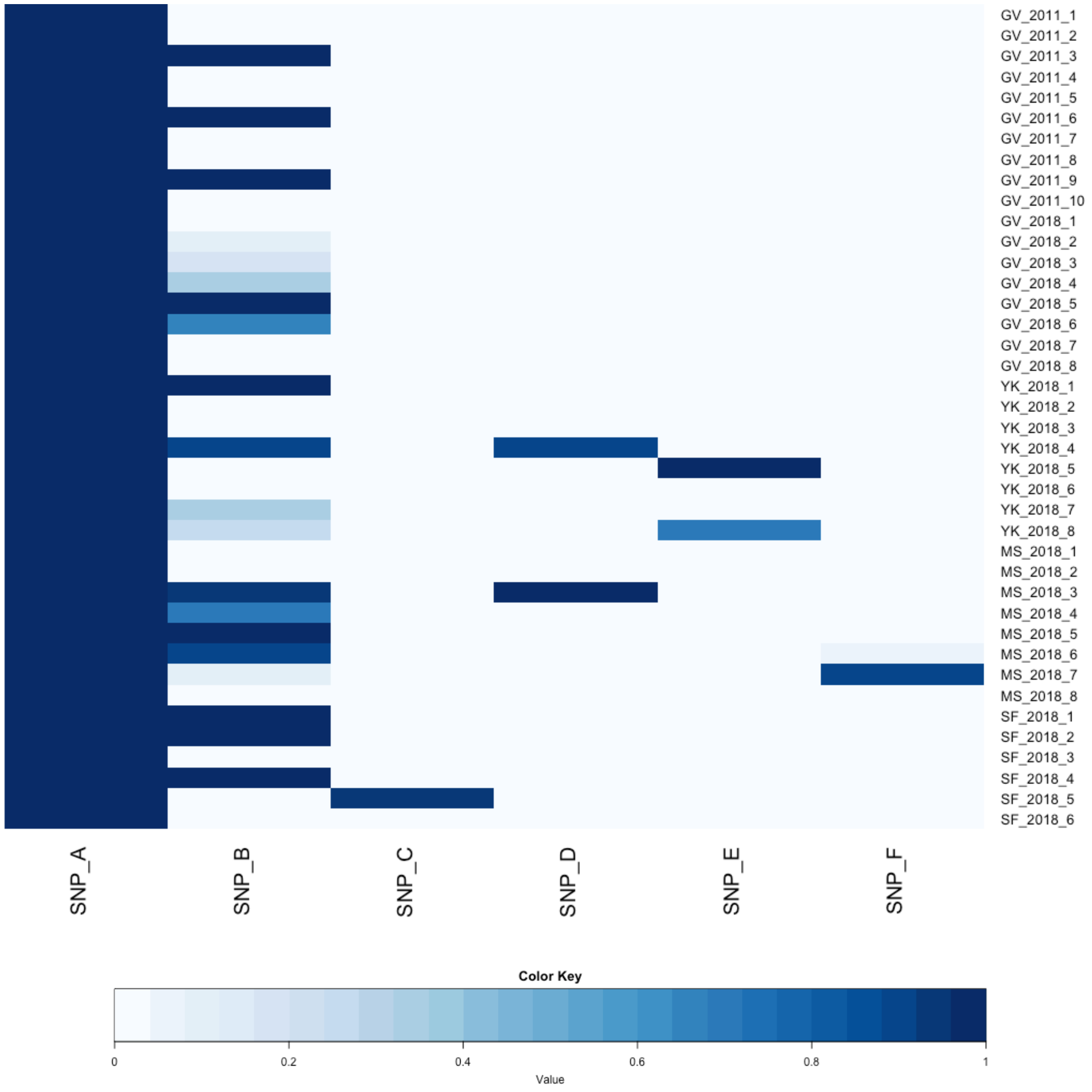

S2 Fig. Identified SNP frequencies across all genomes sequenced. Heatmap showing proportion of reads that mapped to the alternate SNP alleles for each genome sequenced as identified by LoFreq V2. 1. 3. 1. Rows correspond to individual samples, and columns correspond to the six SNPs identified.

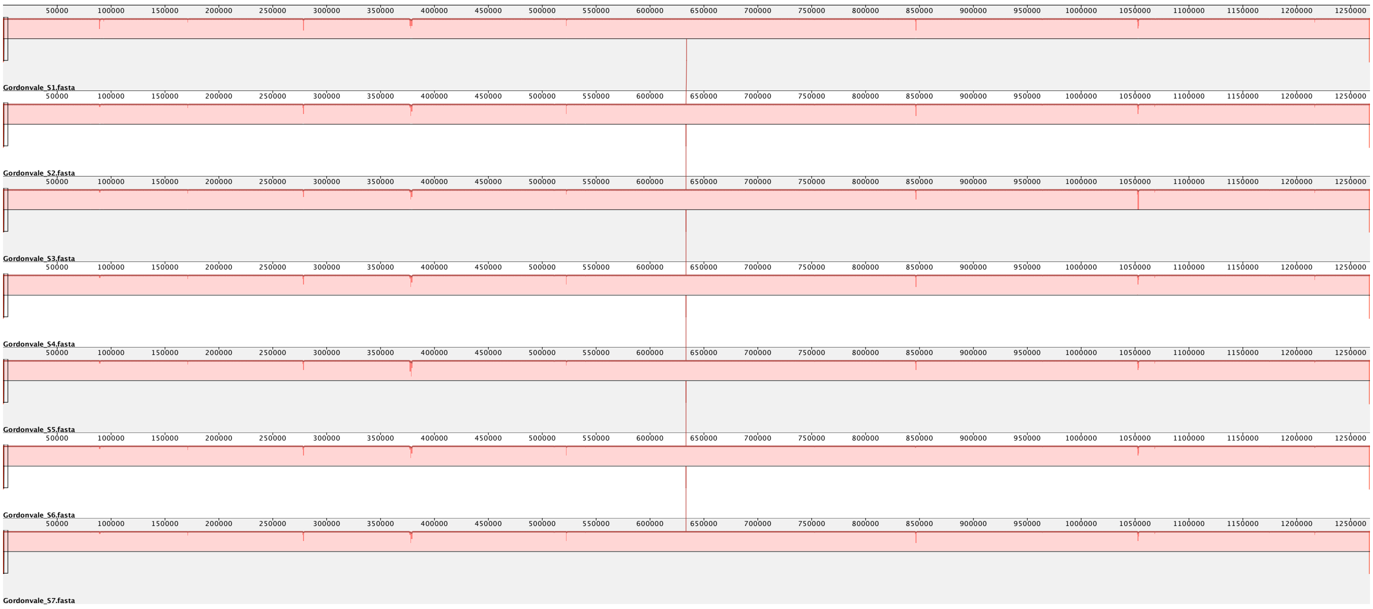

S3 Fig. Genome alignments of the 7 resolved *w*Mel genomes from Gordonvale, Cairns, in reference to the *D. melanogaster w*Mel reference genome. Each red coloured region is a locally collinear block, a region without rearrangement of homologous backbone sequence. The figure was generated by the Mauve rearrangement viewer.

A)
SNP_A Position 1097797
Reference=T, Alternate=A

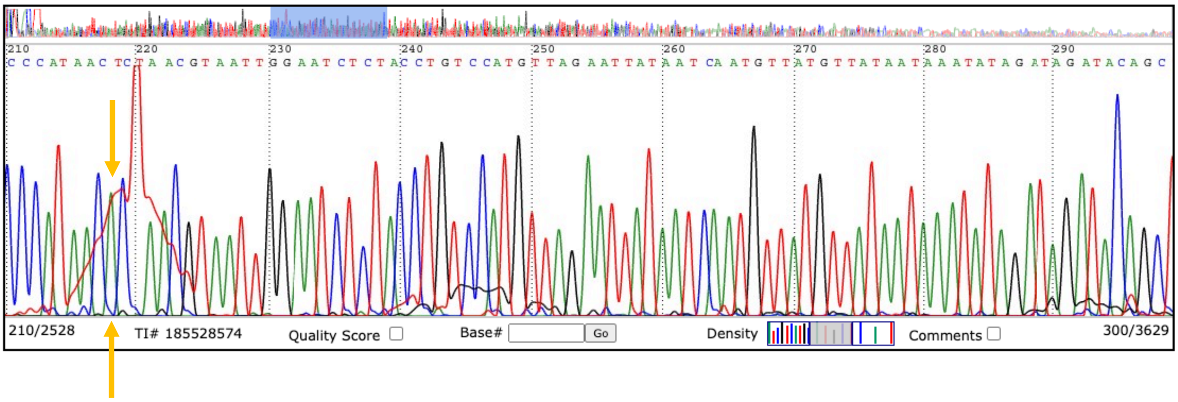

B)
Position 1006081
Reference=GT, Alternate=GTT
Reverse strand

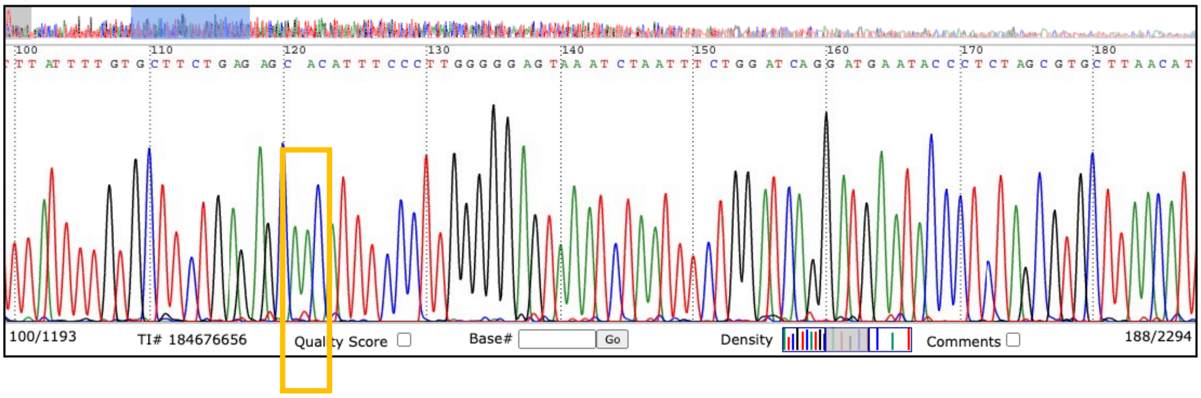

C)
Position 1020475
Reference=CTTTT, Alternate=CTTTTT
Reverse strand

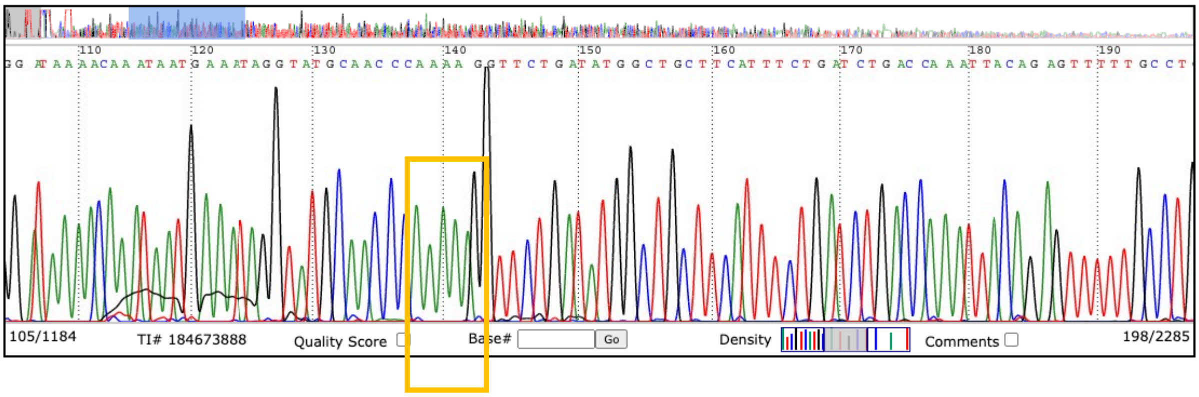

D)
Position 1094458
Reference=GTT, Alternate=GTTT
Alternate strand

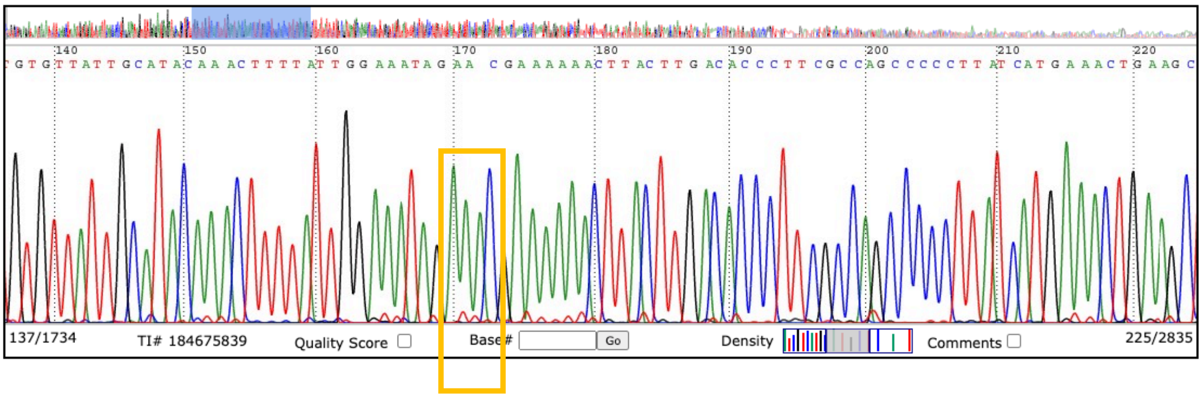

E)
Position 1103468
Reference=ATTTT, Alternate=ATTT

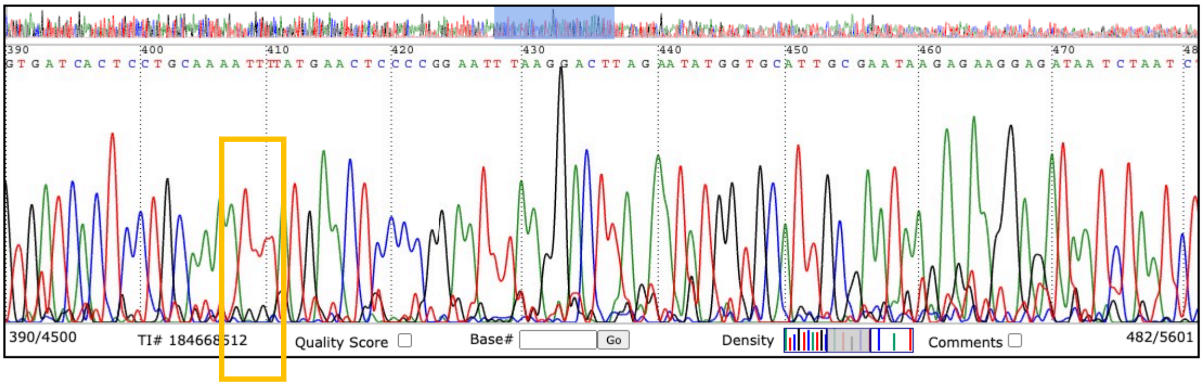

F)
Position 1161850
Reference=GTTTTT, Alternate=GTTTTTT
Reverse strand

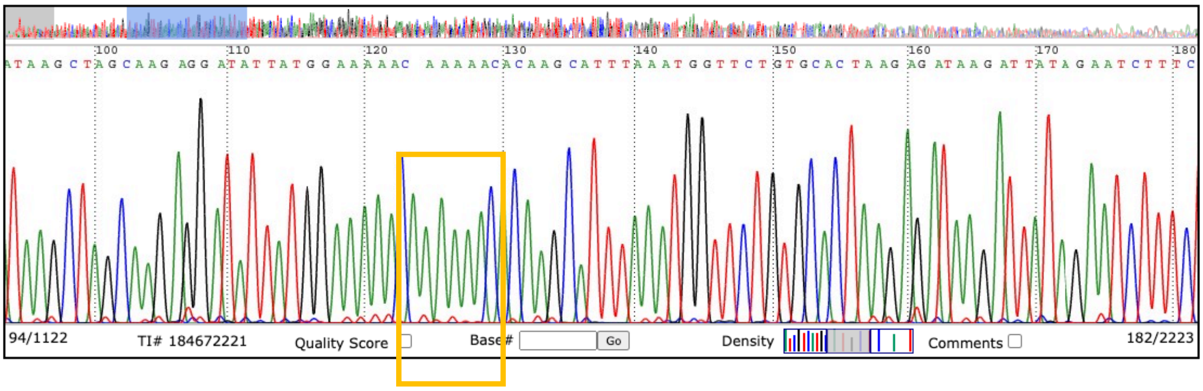

G)
Position 1163170
Reference=GC, Alternate=G

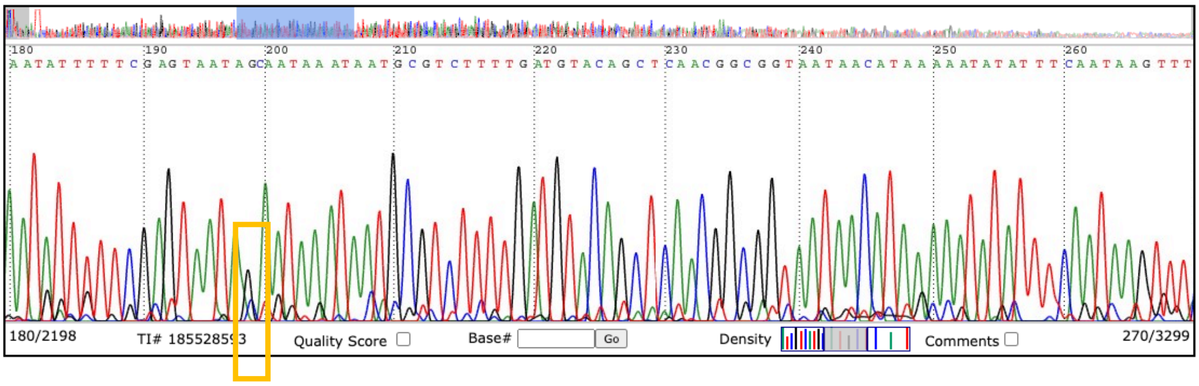

H)
Position 1177853
Reference=ACC, Alternate=AC
Reverse strand

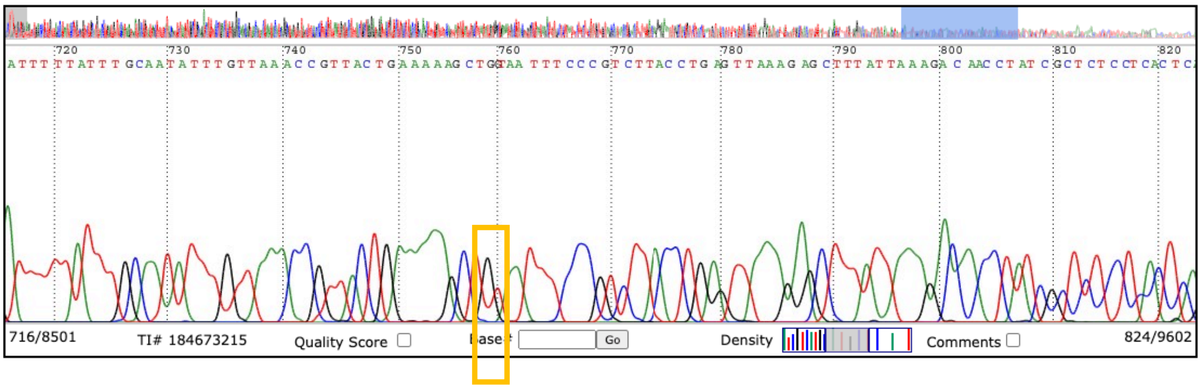

S4 Fig. *w*Mel reference sequence trace images for polymorphisms identified as possible errors. Images of sequence traces from the *w*Mel reference genome highlighting A) SNP, or B-H) indel differences identified to be potential reference sequence errors. Areas of interest are indicated with yellow arrows or boxes. Trace files can be found at: https://www.ncbi.nlm.nih.gov/Traces/trace.cgi?&cmd=retrieve&val=species_code%3D%22WOLBACHIA%20ENDOSYMBIONT%20OF%20DROSOPHILA%20MELANOGASTER%22&retrieve=Submit.

S1 File. Table of low frequency SNP variants for 2018 *w*Mel genomes. Variants were calculated by LoFreq using parameters set out in Methods section. Sample ID indicates the collection suburb (Gordonvale = G, Yorkeys Knob = Y, Mount Sheridan = M, Smithfield S) and numeral relating to sample title.
